# What Is Helpful and What Is Challenging for the Caregivers of Young People Receiving Interventions to Prevent Suicide? Caregivers’ Perspectives—A Rapid Scoping Review

**DOI:** 10.3390/children10111801

**Published:** 2023-11-13

**Authors:** Grace Branjerdporn, Ferrell Erlich, Karthikeyan Ponraj, Laura K. McCosker, Sabine Woerwag-Mehta

**Affiliations:** 1 Mental Health and Specialist Services, Gold Coast Hospital and Health Service, Southport, QLD 4215, Australia; laura.mccosker@griffithuni.edu.au (L.K.M.); sabine.woerwagmehta@health.qld.gov.au (S.W.-M.); 2 Faculty of Health Sciences and Medicine, Bond University, Robina, QLD 4226, Australia; ferrell.erlich@student.bond.edu.au (F.E.); karthikeyan.ponraj@student.bond.edu.au (K.P.); 3 School of Medicine and Dentistry, Griffith University, Nathan, QLD 4111, Australia

**Keywords:** youth, adolescent, child, suicide, caregiver, parent, guardian, intervention, prevention, perspective

## Abstract

(1) Background: Suicide is a leading cause of death among young people. Preventing suicide in young people is a priority. Caregivers play a vital role in ensuring interventions for young people experiencing suicide ideation and/or attempts are implemented, and that they are maintained over time. Despite this, little is known about what caregivers find helpful and challenging in relation to suicide prevention interventions. This rapid scoping review is the first to address this gap. (2) Methods: Searches were completed on six electronic databases using keywords relating to ‘suicide prevention, ‘young people’, and ‘caregivers’. Ten studies—using both qualitative and quantitative methods, and involving >1400 carers from the United States and Europe—were selected for inclusion. (3) Results: The review shows that caregivers value interventions that are delivered by non-judgmental clinicians, that are suitable to the particular needs of their child, that are available when needed, and that support their confidence and communication. Caregivers experience difficulties with interventions that require their attendance at specific times, and that fail to recognize and/or address their own mental health needs. (4) Conclusions: The findings can be used to inform and improve the intervention design, with the aim of improving outcomes for caregivers and young people.

## 1. Introduction

### 1.1. Suicide in Young People

Globally, suicide is the fourth leading cause of death among young people [1]. The rate of suicide in young people aged 10–19 years is estimated at 3.77/100,000 [2]. This is an increase from the previous decade, where rates were estimated at 0.94/100,000 for girls and 1.52/100,000 for boys [3]. In the United States, the rate of suicide in young people may be as high as 11.0/100,000 [4]. Far more young people experience suicidal ideation: one meta-analysis estimates that, globally, 22.6% of young people have experienced suicidal ideation [5]. Since the COVID-19 pandemic, the rate of suicide ideation and attempts among young people has increased in many regions, perhaps due to cumulative stress, increased social isolation and technology use, and a reduction in access to mental health services [6].

There are a number of risk factors associated with suicide behavior in young people. Typically, suicide behavior is underpinned by a complex intersection of factors. Mental disorders, including (but not limited to) those involving depression and anxiety, are strong predictors of suicide behavior [7]. Being bullied, having no close friends, poor family functioning, and having low self-esteem are also strong predictors [8,9,10]. Suicide bereavement is another significant predictor [11]. Conversely, strong relationships with family, peers, and other adults (e.g., teachers, mentors, etc.) are significant protective factors [12].

### 1.2. Interventions to Prevent Suicide

Preventing suicide (and self-harm) in young people is a priority. Most high-income countries have a national suicide prevention strategy, and many of these include a specific focus on young people [13]. There are a range of evidence-based interventions aimed at preventing suicide in young people [14]. These typically include pharmacotherapy and various types of psychotherapy, often in combination. Psychotherapy may be delivered face to face, but web-based programs are increasing in popularity [15]. Usually, clinicians also work with young people and their caregiver/s to develop and implement a safety or management plan, which aims to keep a young person safe as other interventions are being implemented [16,17].

Evidence from systematic reviews about the safety and efficacy of interventions to prevent suicide in young people is variable [13,14,18,19,20]. This reflects the variable quality of much of the primary research on the topic. However, these systematic reviews generally support interventions that consider the young person in their broader environment, which include the people significant to the person, and which require a sustained commitment over time. However, the exact nature of these interventions can vary significantly between settings.

### 1.3. The Role of Caregivers

‘Caregivers’ are people who provide informal, unpaid care and support to a young person during a period of psychological distress [21]. Caregivers are typically biological parents, but may also include step, adoptive, and foster parents, grandparents, other relatives, and legal guardians [22]. The types of care and support caregivers provide to young people are highly situation-specific, but they might involve practical support (e.g., facilitating access to interventions, reducing access to lethal means, etc.), emotional support, monitoring and observation, communication, safety planning, education, and advocacy, in addition to other functions.

Caregivers play a vital role in ensuring interventions for young people experiencing suicide behaviors are implemented, and that they are maintained over time. Caregivers are often experts in the experiences and needs of the young person they care for. Young people are, in many cases, most likely to disclose suicide behaviors to their caregivers [23]. Young people are also often dependent on their caregivers as a primary source of practical and emotional support, and caregivers are therefore well-positioned to observe and monitor them. Many interventions are delivered in the community (and, in the case of safety plans, often in the young person’s home environment); these require long-term commitment which, for younger people at least, must be reinforced by a caregiver. Young people may be more likely to accept professional help when their caregivers are supportive of it [24]. In young people, connectedness with a caregiver is a significant predictor of reduced suicide behavior [25,26,27].

### 1.4. Caregivers’ Perspectives of Interventions to Prevent Suicide

Despite the importance of caregivers in supporting young people with suicide behaviors, little is known about what caregivers find helpful and challenging in relation to suicide prevention interventions. This means that caregivers’ perspectives are likely often overlooked in intervention design, perhaps to the detriment of the young people they support. This also means that caregivers may not be as well-supported in these interventions as they might be.

There are two systematic reviews about the experiences of the caregivers of young people who experience suicidality [28,29]. These both consider, in part, caregivers’ perspectives of the information and support they receive. There is also a third systematic review which considers caregivers’ perspectives of family-based therapies specifically [30]. However, ours is the first review to examine caregivers’ perspectives of the suicide prevention interventions provided to young people. We aim to identify what caregivers find helpful, and what they find challenging, in relation to suicide prevention interventions. In doing so, the review will present evidence to inform, and ultimately improve, intervention design, with the aim of improving outcomes for caregivers and young people.

## 2. Materials and Methods

### 2.1. Rapid Scoping Review

This is a rapid scoping review. Rapid scoping reviews simplify, shorten, or even eliminate one or more of the components of a systematic review, with the aim of presenting evidence in a more timely manner [31]. This review was completed by a team of clinician-researchers working in a youth mental health service in Australia. We required rapid evidence about caregivers’ perspectives of suicide interventions to enable us to better understand the feedback we received from caregivers about the interventions offered by our service. Caregivers indicated in routine feedback that they were, and were not, satisfied with different interventions. We undertook this review to identify the possible reasons why, to inform intervention design. As a literature review, this study was exempt from human research ethics clearance.

### 2.2. Eligibility Criteria

To develop the eligibility criteria for this review, the population–exposure–outcome (PEO) framework was used. The ‘population’ was any person, of any age, who was a caregiver of a young person (≤17 years of age) receiving any intervention/s aimed at preventing suicide. Biological, step, adoptive, and foster parents, grandparents, other relatives, and legal guardians were all considered to be caregivers. The ‘exposure’ was the intervention/s received by the young person. These interventions may or may not have directly involved the caregiver. Interventions must have been delivered in a healthcare setting (e.g., an emergency department, hospital inpatient setting, and/or outpatient/community service setting). The ‘outcomes’ included the caregiver’s experiences and perspectives about the child’s care.

Studies with no explicit mention of the suicide prevention intervention/s received by the young person were excluded. Studies with interventions delivered in non-healthcare settings, including school settings, were excluded. Additionally, studies that examined only the experiences and perspectives of the child and/or of the healthcare professional/s were excluded. Studies using any methodology (quantitative, qualitative, or mixed methods) were considered for inclusion. Only the literature published in a peer-reviewed academic journal, in the English language, and in full text were considered. The searches were not limited by date or region.

### 2.3. Information Sources

Searches were completed on six electronic databases: CINAHL, Embase, MEDLINE, PsycINFO, PubMed, and Scopus. These databases were selected because they are the largest sources of health and social science literature. Searches were conducted until 29 December 2022. Searches used three groups of keywords, relating to ‘suicide prevention, ‘young people’, and ‘caregivers’. Subject headings, parentheses, truncation, and Boolean operators were used where relevant. Table 1 presents an example of the search strategy used on PubMed.

### 2.4. Selection

The results of each search were exported into EndNote V20 (Clarivate, Ann Arbor, MI, USA), and then imported into Covidence (Veritas Health Information, Melbourne, Australia). Duplicates were removed through automatic and manual processes. The literature was screened according to title/abstract and then, for the remaining items, according to the full text. At each step, two reviewers (FE, KP) completed the screening, and two other reviewers (GB, SWM) mediated disagreements where needed. A fifth reviewer (LM) completed a final check of the studies.

### 2.5. Data Extraction

Electronic data extraction tables were developed by the reviewers. The data extracted included information about the following: (a) the study and participant characteristics, (b) the intervention/s received by the young person, and (c) caregivers’ experiences and perspectives of that intervention. Data extraction was completed separately by two reviewers (FE, LM), and checked by a third reviewer (KP). Two other reviewers (GB, SWM) mediated disagreements if needed.

### 2.6. Quality Assessment

The studies selected for inclusion were assessed using the Quality Assessment with Diverse Studies (QuADS) tool. This tool is designed to evaluate the quality of studies completed using a variety of different methods, across a range of health- and social-care-focused disciplines [32,33]. It consists of 13 items, which are scored as 0 = item has no mention at all, 1 = item is inadequately reported, 2 = item is adequately reported, and 3 = item is explicitly reported. The scores for each of the 13 items are then added to generate a total score. Each paper was independently evaluated by two reviewers (FE, KP). These reviewers then discussed and agreed upon a total score. Studies were not excluded from the review based on low total scores, due to the limited number of eligible papers.

### 2.7. Data Synthesis

Because the studies considered for this review used diverse methodologies, and reported findings both quantitatively and qualitatively, a narrative synthesis was undertaken. Data in relation to each of the items extracted were organized thematically, critically examined for similarities and differences, and objectively reported using descriptive text [34].

## 3. Results

### 3.1. Searches

**Figure 1:**
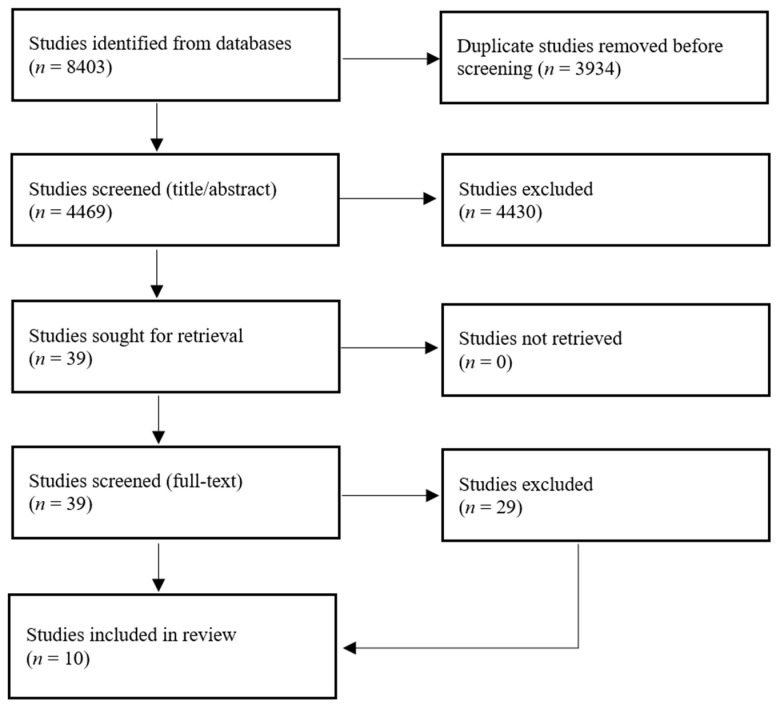
PRISMA diagram showing the literature selection process.

The selection process is illustrated in Figure 1, as a Preferred Reporting Items for Systematic Reviews and Meta-Analyses (PRISMA) diagram. A total of 8403 studies were retrieved through the database searches. In total, 3934 duplicates were removed, leaving 4469 unique studies for screening. During the screening of titles/abstracts, 4430 studies were removed. The remaining 39 studies were retrieved in full text. Twenty-nine of these studies were excluded, primarily because they involved the wrong intervention (*n* = 15) and/or the wrong population (*n* = 10). Ten studies were selected for inclusion [35,36,37,38,39,40,41,42,43,44].

### 3.2. Study and Participant Characteristics

Table 2 presents the characteristics of the included studies, and the study participants. There were nine quantitative studies [35,36,37,38,39,40,41,42,43] and one qualitative study [44]. Most of the studies (*n* = 7, 70.0%) were conducted in the United States [35,37,38,39,40,41,43]. There were two studies from the United Kingdom [36,42,44], and one from Sweden [42]. The studies involved a range of caregivers, including biological, step, adoptive, and foster parents, grandparents, other relatives, and legal guardians. Three of the papers did not explicitly identify the type/s of caregivers participating, but simply referred to them as ‘caregivers’, ‘parents’, and/or ‘guardians [37,41,43]. Sample sizes ranged from 32 caregivers [37] to 832 caregivers [36]. Two of the studies reported the number of families participating without noting the number of caregivers [41,43], and one other study did not specify how many caregivers participated [42]. Many of the studies involved >1 caregiver for each young person receiving intervention/s.

### 3.3. Intervention Characteristics

Table 3 presents the characteristics of the suicide prevention intervention/s received by the young people in the studies. Most of the studies (*n* = 6, 60.0%) reported on the interventions delivered, at least in part, in an inpatient hospital setting [35,36,37,39,43,44]. Six of the papers looked at specific interventions from a single provider; these included family therapy [36] and bundled interventions (Motivational Interviewing [MI]-SafeCope [37], a specialized emergency department program [40], Family-Based Crisis Intervention [FBCI] [41], Intensive Contextual Treatment for Self-Harm [ICT] [42], and Coping Long Term with Active Suicide Program—adolescents [CLASP-A] [43]). Often, these studies compared the intervention with treatment, as usual. The remaining papers involved young people receiving ≥1 treatments from ≥1 providers [35,38,39,44]. The interventions may or may not have involved the caregiver.

Common interventions received by the young people included individual psychotherapy, family therapy, and pharmacotherapy. Common features of these interventions included that they were conducted over multiple sessions, often with more frequent initial appointments; that they focused on safety planning and associated activities to keep the young person safe; that they included educational and motivational components; that they aimed to facilitate and/or improve communication between the young person and their caregiver/s; and that they included follow-up with the young person and/or their caregiver/s over time. The interventions were universally delivered by trained health and/or mental health professional/s.

### 3.4. Caregivers’ Experiences and Perspectives

Table 3 also presents findings from the study, in relation to caregivers’ experiences and perspectives of the intervention their child received. Two broad themes were identified:

Theme 1: What caregivers find helpful.

The caregivers identified a variety of different types of interventions that may be ‘helpful’ for their child at risk of suicide, including individual psychotherapy [35], family therapy [36], pharmacotherapy [35], and bundled interventions involving ≥1 of these (and other) components. In general, caregivers were accepting of a range of interventions [38]. However, caregivers’ positive attitudes about interventions might be encouraged through specialized programs that include education from a health professional aimed at clarifying their treatment expectations [40]. Caregivers valued the ‘correct’ intervention to suit the particular needs of their child, access to the right context of care (e.g., a children’s rather than an adult’s hospital ward), and intensive support early on (including from a crisis team, where relevant) [44].

A small number of the studies explored why caregivers consider certain interventions helpful. They consider interventions that promote the caregivers’ readiness to encourage a young person’s safety plan [37], support their self-efficacy to engage in suicide prevention activities generally [38], or otherwise empower them in relation to suicide prevention [41] to be particularly important. These interventions often involved teaching practical strategies (e.g., what to do, where to access information, etc.), via educational resources such as talks, websites, and leaflets, etc. [38,44]. Higher caregiver self-confidence is positively correlated with higher engagement in and compliance with the young person’s discharge plan [38,39].

None of the studies were able to further correlate these outcomes with reduced suicide risk, typically because of the small numbers of suicides among the participating young people. However, this is a logical conclusion. It is certainly an important area for future research.

The studies identified that caregivers’ perspectives of the helpfulness of an intervention may shift upwards and downwards over time [35,36]. Further, over time, caregivers may perceive interventions as being equally, more, and less helpful than the young person receiving the intervention [35,37,42,43]. Such discrepancies in perceptions may cause tension or conflict.

Finally, caregivers also identified the communication processes embedded in interventions as being helpful. This included communication with healthcare professionals: caregivers valued professionals who listened to, involved, regularly updated, and provided feedback to them, and who were open with them about their child’s care [44]. Parents valued professionals who were non-judgmental, caring, sensitive (including during assessments), took them and their child seriously, and took time to build rapport [44]. It also included communication with their child: carers valued interventions that promoted healthier interactions with their child [42].

Theme 2: What caregivers find challenging.

The caregivers also identified features of interventions they found challenging. Attendance and engagement were sometimes difficult, as caregivers frequently juggled multiple life stresses. In one study, caregivers only attended 75% of the scheduled sessions, whereas young people attended 90%, and attendance declined over time [43]. In another study, 79% of caregivers considered themselves to be engaged in their child’s discharge plan, although only 44% of young people considered their caregiver to be engaged [38]. In both caregivers and young people, less-positive family relationships were correlated with greater non-attendance and disengagement [40]. Caregivers valued interventions with telephone check-ins; these were convenient, and they provided an opportunity to pause and reflect during the busy day [43].

Caregivers also found the limitations associated with the healthcare systems responsible for delivering the interventions challenging. Many were uncertain about how to best support their child and desired increased clinical support, particularly early on, but this was not always available [44]. Similarly, caregivers valued prompt access to care for their child, such as interventions involving on-call therapists, but again this was not always available [40,44].

In one of the studies, half (51%) of the participating caregivers experienced clinically significant distress related to their child’s condition [38]. Caregivers reported such stressors as seeing the child sad/scared, knowing the child is hurting/in pain, and feeling helpless about the child’s condition [38]. Interventions involving specialized psychotherapeutic support might benefit caregivers [40,42] and also increase their satisfaction [41]. Caregivers also identified that parent (peer) support groups, and mental health support for themselves, were important [44]. However, it is unclear if any of the interventions offered this type of support.

### 3.5. Quality Assessment

Table 4 presents the quality assessment of the studies. Across the studies, the mean QuADS score was 32.3 points (out of a maximum possible 39 points, ranging from 24 points [39] to 38 points [40]). The most poorly scored criterion was the consideration of stakeholders’ input into the research design: 5 of the 10 studies (50.0%) scored zero for this criterion [35,36,39,42,43]. It is possible that stakeholders’ input was not explicitly noted in the papers. However, and particularly in the field of mental health, research that is co-designed, co-produced, and co-facilitated, allowing people with lived experiences to contribute, is vital to quality outcomes.

## 4. Discussion

This rapid scoping review is the first to identify what caregivers find helpful and challenging in relation to suicide prevention interventions for the young people they support. It included ten studies involving >1400 carers from the United States and Europe. It identified a number of features of interventions that caregivers perceive to be helpful, and others they find challenging. These findings can be used to inform, and ultimately improve, intervention design.

### 4.1. Interventions That Are Delivered in the Right Context

This review suggests that caregivers are, perhaps, less concerned about the specific type/s of interventions their young person receives (i.e., pharmacotherapy versus psychotherapy), and more about the context in which they are delivered. Specifically, caregivers value interventions that are delivered by non-judgmental clinicians, that are suitable to the particular needs of their child, and that are available when needed. Broader research agrees that caregivers value empathetic, validating, and non-judgmental approaches to intervention delivery [45]. A poor encounter with a health service—such as an experience involving stigma [46], blame [47], and/or guilt [48]—might discourage caregivers from seeking further help [49], undoubtedly leading to poorer outcomes both for themselves and for their young person. It is vital that the staff providing interventions are properly trained to communicate with caregivers.

### 4.2. Interventions That Include Psychological Support for Caregivers

The literature further agrees with our findings, that caring for a young person experiencing suicide ideation and/or attempts is an immense burden on caregivers [29]. In other research, caregivers describe being “bewildered” or blindsided [50], dismissive of their own needs [49], so focused on their child that other relationships break down [51], and even distressed to the extent that they too consider suicide [52,53]. It is interesting to note the research suggesting that suicidal ideation in young people is associated with depression in caregivers [54].

When caregivers are supported, they are better able to care for their child [45]. Our review identified a variety of ways that caregivers might be supported. The broader literature agrees that counselling and other psychiatric services for caregivers, delivered with as well as separately from the young person, are key [38,55]. Interventions involving follow-up calls and respite care, offering caregivers support and a break from the demands of their caregiving role, might also be beneficial [56,57]. However, this review identified that mental health services in many regions are often stretched, and that interventions for young people—let alone interventions catering specifically for caregivers—may not be readily available.

In the absence of support from the healthcare system, caregivers describe seeking support “from other parents, from books, from the internet, [and] from research papers” [52]. Providing these resources to caregivers is an essential consideration. Research shows that caregivers value resources that are user-friendly, use non-clinical language, and have simple layouts [47]. Another option for supporting caregivers might be peer support; indeed, research indicates that caregivers are very willing to help their peers [47,52]. Self-care is also key [47], but skills may need to be taught and encouraged by clinicians. These simple interventions might relieve caregivers’ uncertainties about their ability to support their young person, and avoid them feeling as if they need to ‘hold on’ waiting for help [58,59]. Ultimately, they may improve caregivers’ ability to support the young person in participating in interventions.

### 4.3. Interventions That Are Flexible

This review found that many caregivers consider attendance at, and sustained engagement in, suicide prevention interventions to be difficult. Caregivers may experience a variety of competing demands, including the following: caring for other family members, maintaining employment, and running a household, etc. Flexible interventions, including those listed in the previous section, are therefore an essential consideration. However, again, it may be difficult for over-burdened healthcare systems to offer such interventions, including, for example, routine psychotherapy appointments after work hours or on weekends [28]. In the broader literature, caregivers frequently describe needing to take time off work to focus on caring for their young person [28,55]. Providers might consider strategies such as online sessions, or separate sessions for young people and caregivers, to facilitate caregivers’ involvement [30]. Interventions delivered using communication technologies, and which can be accessed flexibly at times and locations convenient to caregivers, might also be effective [60].

### 4.4. Interventions That Increase Caregivers’ Confidence and Self-Efficacy

This review suggests that interventions which support caregivers’ confidence and self-efficacy to engage in suicide prevention activities are important, regardless of what specifically those interventions involve. The broader literature agrees that caregivers are often ill-equipped, at least initially, to provide the support that their young person requires [28,61]. Studies describe successfully incorporating psychoeducational components for caregivers into suicide prevention interventions for young people [62]. Other interventions include specific training activities for caregivers, such as multi-family skills training, which might include telephone coaching as required [63]. Where healthcare services are over-burdened and unable to deliver such interventions, even an instructive conversation with a healthcare provider—for example, during the process of safety planning—might be beneficial.

### 4.5. Interventions That Facilitate Caregivers’ Communication

Broader research also agrees with our findings that caregivers value interventions that promote improved communication with the young person they support [64], again regardless of what specifically those interventions involve. Difficulties in the relationship with care-givers, and withdrawal from that relationship, are common in young people with suicidal behaviours [65]. Poor communication with caregivers is a significant predictor of self-harm in young people [66]. However, communication about suicide is difficult, as explained by one caregiver: “When you do not talk about it you can imagine or pretend it does not exist, so you don’t need to do anything [about] it” [24]. Caregivers value techniques that address how to talk with young people about how they are feeling and what they need [28]. Again, improved communication might better equip caregivers with the tools needed to support engagement with interventions.

### 4.6. Limitations

It is important to consider the findings of this review in the context of its limitations. The review identified relatively few studies, though it is important to acknowledge that studies, both in the mental health field and generally, tend to overlook informal caregivers [22]. In comparison to similar reviews in adult populations [22], this review identified comparatively fewer qualitative studies. This study also included a less diverse field of caregivers than in other studies (where ‘caregivers’ might have included siblings, friends, and neighbours, etc. [28]). All of the studies included were undertaken in high-income countries, and the findings may not translate to other settings. Further, the studies were limited to those published in English and available on six select databases; others may have been missed. As a rapid scoping review methodology was used, it is likely that some studies were overlooked.

It is important to acknowledge the paucity of literature about caregivers’ perceptions of suicide prevention interventions, and the correlation with suicide risk in the young people they support. It is reasonable to assume that when caregivers perceive an intervention positively, this will result in greater and more sustained engagement, and positive outcomes for their young person. This is an interesting area for future research. Another interesting area for future research is to consider the perspectives of caregivers directly involved, and those not directly involved, in interventions. As emphasized earlier, stakeholder input into research design is another vital consideration for future research.

## 5. Conclusions

This rapid scoping review is the first to identify what caregivers find helpful and challenging in relation to suicide prevention interventions for the young people they support. The review shows that caregivers value interventions that are delivered by non-judgmental clinicians, that are suitable to the particular needs of their child, that are available when needed, and that support their confidence and communication. They experience difficulties with interventions that require their attendance at specific times, and that fail to recognize and/or address their own mental health needs.

These findings can be used to inform, and ultimately improve, the design of suicide prevention interventions for young people. Where interventions are designed to harness the factors that caregivers find helpful, and to avoid or address the factors they find challenging, this is likely to improve caregivers’ willingness and ability to participate in them. It is, therefore, also likely to improve outcomes for the young people these caregivers support, including perhaps reducing suicidal ideation and attempts. Future research is important in further understanding how to support caregivers, and young people, through intervention design.

## Figures and Tables

**Table 1:** Example search strategy for the PubMed database.

Search Strategy	
((Suicide prevention) OR (Suicide intervention)) AND ((Child*) OR (Youth) OR (Adolescen*) OR (“Young people”) OR (Teenager*) OR (Paediatric) OR (Pediatric)) AND ((Carer) OR (caregiver) OR (parent*) OR (Foster carer) OR (Mother) OR (Father))	
Subject Category	Search Terms
Suicide prevention	suicide screen* suicide prevention suicide intervention suicide risk screening suicide assessment suicide risk assessment self-harm suicidal attempt
Youth and adolescents	child* youth adolescen* “young people” teenager* paediatric paediatric
Caregivers	carer caregiver parent* foster carer mother father

The wildcard symbol (“*”) searches for all variations of the preceding term.

**Table 2:** Characteristics of the included studies and study participants.

Citation	Country	Caregiver Examined					Child Age (Years)	Child Gender		Carer Sample Size	Setting		
		Mother/Step	Father/Step	Adoptive/Foster	Other Relatives	Not Reported		Male	Female		Emergency Department	Inpatient Hospital	Outpatient/ Community
Quantitative Studies													
Burns et al., 2008 [35]	United States	X	X	X			13–18	X	X	132		X	X
Cottrell et al., 2018 [36]	United Kingdom	X	X	X	X		11–18	X	X	832		X	
Czyz et al., 2019 [37]	United States					X (“parents”)	13–17	X	X	32		X	
Ewell Foster et al., 2022 [38]	United States	X	X		X		10–17	X	X	118	X		
King et al., 1997 [39]	United States	X	X				13–17	X	X	61		X	
Rotheram-Borus et al., 1996 [40]	United States	X					12–18	X		140	X		X
Wharff et al., 2019 [41]	United States					X (“parents”, “caregivers”, “guardians”)	13–18	X	X	112 families	X		
Wijana et al., 2018 [42]	Sweden	X	X				13–19	X	X	Not specified			X
Yen et al., 2019 [43]	United States					X (“parents”)	12–18	X	X	17 families		X	
Qualitative Studies													
Stewart et al., 2018 [44]	United Kingdom	X	X	X			9–21	X	X	37	X	X	X

**Table 3:** Intervention characteristics and caregivers’ perspectives.

Citation	Aim(s)	Intervention Characteristics	Caregivers’ Perspectives
Quantitative studies			
Burns et al., 2008 [35]	To identify what adolescents who have attempted suicide, and their caregivers, consider ‘helpful’ in relation to mental health treatment, and to identify factors correlated with treatment compliance and suicidality outcomes	Participants might have received one or more treatments. The most common treatments were individual psychotherapy, family therapy, pharmacotherapy, and inpatient hospital treatment	Most caregivers considered individual psychotherapy helpful, but this decreased over time (60.3%, 58.8%, 52.6%, 45.2%); adolescents considered individual psychotherapy more helpful Fewer caregivers considered pharmacotherapy helpful, but this increased slightly over time (21.5%, 34.7%, 44.1%, 41.4%); adolescents considered pharmacotherapy more helpful When parents perceived their child’s treatment to be helpful, this was a significant predictor of the child’s compliance
Cottrell et al., 2018 [36]	To assess the clinical and cost-effectiveness of family therapy compared with treatment as usual for young people who have engaged in self-harm +/− suicidal ideation/attempt	Family therapy sessions with trained therapists: 8× sessions delivered over 6 months at approx. monthly intervals (with more frequent initial appointments), 1.25 h duration per session	Caregivers participating in family therapy reported significant improvements in their child’s emotional problems, peer problems, and internalising sub-scores (12–18 months), and in their child’s conduct problems and externalising sub-scores (18 months) (Strengths and Difficulties Questionnaire) Caregivers participating in family therapy reported significant improvements in family functioning (12 months); however, this decreased over time (McMaster Family Assessment Device) When health benefits to caregivers (as well as participants) are considered, family therapy may be a cost-effective intervention
Czyz et al., 2019 [37]	To determine the feasibility and acceptability of a motivational interview-enhanced safety planning intervention (MI-SafeCope) for teenagers hospitalized due to suicide risk	The MI-SafeCope intervention includes these components: (a) Establishing a personalized safety plan for the adolescent, (b) employing motivational interviewing strategies to strengthen the adolescent’s motivation and self-efficacy in relation to the plan (through individual meetings, family meetings, and follow-up calls), (c) enlisting the parents’ support to encourage safety plan use	Parents participating in the MI-SafeCope intervention were highly satisfied with it (3.71/4.00), and most would recommend it to a friend (3.82/4.00); the participating adolescents reported similar satisfaction/recommendation outcomes Parents participating in the MI-SafeCope intervention considered it very helpful (9.12/10.00); adolescents also considered it helpful, but somewhat less-so than their parents (7.50/10.00) Parents participating in the MI-SafeCope intervention reported significantly higher readiness to encourage safety plan use
Ewell Foster et al., 2022 [38]	To determine caregiver-level factors, and their correlation with behavioural engagement, in caregivers of youth presenting to an emergency department with suicidal ideation/attempt	Standard emergency department care (risk assessment, safety planning, lethal means counselling, treatment linkage)	Parents reported their desired outcomes from the emergency department as: wanting inpatient hospitalisation for their child (58.5%), wanting educational resources (e.g., on mental health, risk management, etc.) (56.0%), wanting an outpatient referral (41.5%), and wanting a medication script/adjustment (41.5%) Parents were fairly accepting about psychological treatment Parents had moderately high self-efficacy or confidence in their ability to engage in suicide prevention activity (75.24/100.00) Half (51.3%) of parents experienced distress (i.e., some level of depression and/or anxiety) in relation to their child’s condition Parents reported these key stressors in relation to their child’s condition: seeing the child sad/scared, knowing the child is hurting/in pain, feeling helpless about the child’s condition Parents rated themselves as more engaged in the child’s discharge plan than the child rated their parents; the item with the greatest discordance was participation in safety plan activities (79.2% of parents vs. 43.7% of youth, reporting engagement) The only caregiver-level factor positively correlated with behavioural engagement was parental self-efficacy/confidence
King et al., 1997 [39]	To identify parent and family predictors of compliance with treatment after hospitalization in adolescents with suicidal ideation/attempt	Participants might have received pharmacotherapy, individual psychotherapy, and/or parent guidance/family therapy sessions	Family characteristics were strong predictors of compliance. Lower compliance was associated with higher family dysfunction, fathers who were less involved/affectionate, and mothers with higher depression/paranoia/hostility scores
Rotheram-Borus et al., 1996 [40]	To evaluate outpatient treatment adherence in adolescents who have attempted suicide, and who participate in either standard care or a specialized emergency department program	The specialized emergency department program included staff with training in adolescent suicide, a videotape clarifying families’ treatment expectations, and an on-call family therapist	Mothers participating in the specialised program reported less psychopathology, greater positive attitudes towards treatment There was no significant difference in mothers’ attendance between the two programs; however, adolescents in the specialised program were significantly less likely to drop out Single parents, and parents with adolescents reporting more positive family relations, were less likely to drop out
Wharff et al., 2019 [41]	To examine the efficacy of a Family-Based Crisis Intervention (FBCI), versus treatment as usual, in a hospital emergency department for adolescents with suicidal ideation/attempt	The FBCI involves a single 60- to 90-min session delivered during the emergency department visit. A clinician teaches psycho-education, cognitive behavioural skill building, therapeutic readiness, safety planning, and unified crisis narrative development	Parents participating in the FBCI reported higher levels of family empowerment, and higher levels of satisfaction with the care provided in the emergency department
Wijana et al., 2018 [42]	To determine the effectiveness of an integrated individual and family therapy intervention (Intensive Contextual Treatment for Self-Harm [ICT]) in for adolescents with self-harm and/or suicidal ideation/attempts	The ICT intervention involves twice-weekly meetings for 3 months, delivered at the family home (wherever possible). A therapist teaches adolescents and caregivers effective emotional regulation, functional communication within the family, strategies for attendance of school/other scheduled activities, and maintenance and action planning in case of relapse	Adolescents participating in the ICT intervention reported a reduction in perceived criticism, and a slight increase in perceived emotional involvement, from both of their parents Parents participating in the ICT intervention reported reduced levels of stress/anxiety/depression, making fewer critical remarks to their child and reduced emotional over-involvement Mothers (3.62/4.00) and fathers (3.51/4.00) were highly satisfied with the intervention; adolescents were less satisfied (3.25/4.00)
Yen et al., 2019 [43]	To test the feasibility and acceptability of the Coping Long Term with Active Suicide Program—adolescents (CLASP-A), versus treatment as usual, in adolescents with suicidal ideation/attempts	The CLASP-A program involves a therapist conducting two in-person sessions with the adolescent, one in-person session with the adolescent and their parent, then one telephone session each with the adolescent and their parent. The sessions focus on psycho-education life/safety planning, communication, problem-solving	Parents attended 75% of the sessions, and adolescents attended 90% (open pilot); participation declined over time (pilot) Both parents (3.32/4.00) and adolescents (3.62/4.00) reported being satisfied with the CLASP-A program (quality, service) Parents considered the service an adjunct to other psychiatric care; parents also reported liking the telephone check-ins as these allowed them to stop and reflect about changes over time
Qualitative studies			
Stewart et al., 2018 [44]	To explore the experiences of parents of young people who self-harmed, in terms of seeking support and navigating the healthcare system	Participants might have received one or more treatments. The most common treatments were cognitive and dialectical behaviour therapies	Parents valued health professionals who were non-judgemental, caring, sensitive (including during assessments), took them/their child seriously, and built rapport Parents valued prompt access to care (but this was not always available), access to the right context of care (e.g., children’s wards), intensive support early on (including from a crisis team), the correct treatment (e.g., therapy, medication), and access to practical strategies (e.g., what to do, how to access information, feedback from staff, etc.). They reported web resources, organisations, and leaflets as helpful/supportive Parents considered it important for themselves/their families to be involved in the child’s care. Many were uncertain and wanted clinical support (but this was not always available). Parents found clinicians who listened to, involved, regularly updated, and communicated openly with them to be important. They also reported parent (peer) support groups, and mental health support for themselves, to be important

**Table 4:** Quality assessment of the included studies.

Citation	1. Theoretical or Conceptual Underpinning to the Research	2. Statement of Research Aim/s	3. Clear Description of Research Setting and Target Population	4. The Study Design Is Appropriate to Address the Stated Research Aim/s	5. Appropriate Sampling to Address the Research Aim/s	6. Rationale for Choice of Data Collection Tool/s	7. The Format and Content of Data Collection Tool Is Appropriate to Address the Stated Research Aim/s	8. Description of Data Collection Procedure	9. Recruitment Data Provided	10. Justification for Analytic Method Selected	11. The Method of Analysis Was Appropriate to Answer the Research Aim/s	12. Evidence That the Research Stakeholders have Been Considered in Research Design or Conduct	13. Strengths and Limitations Critically Discussed	Total Score
Burns et al., 2008 [35]	1	3	2	3	2	3	3	3	3	0	3	0	2	28
Cottrell et al., 2018 [36]	3	2	3	3	3	3	3	3	3	3	3	0	3	38
Czyz et al., 2019 [37]	3	2	1	3	2	1	2	3	3	2	2	2	2	28
Ewell Foster et al., 2022 [38]	3	3	3	3	3	3	3	2	3	3	3	3	3	38
King et al., 1997 [39]	3	3	1	3	2	3	3	3	1	0	1	0	1	24
Rotheram-Borus et al., 1996 [40]	2	3	3	3	3	3	3	3	3	3	3	3	3	38
Stewart et al., 2018 [44]	1	3	3	2	3	3	3	3	3	3	3	1	3	34
Wharff et al., 2019 [41]	2	3	3	3	3	3	3	3	3	2	2	2	1	33
Wijana et al., 2018 [42]	3	3	2	1	2	3	3	3	1	2	3	0	3	29
Yen et al., 2019 [43]	3	3	2	3	3	3	3	3	1	3	3	0	3	33

## Data Availability

Not applicable.
